# North Carolina Veterinary Medical Association

**Published:** 1897-12

**Authors:** J. W. Petty

**Affiliations:** Secretary


### NORTH CAROLINA VETERINARY MEDICAL ASSOCIATION.

THE meeting was held at Central Hotel, Charlotte, September 7 and 8, 1897. President Ellis called the meeting to order. Members present: Drs. C. R. Ellis, John Petty, H. G. Bessent, H. F. Bauer, H. B. Murray, J. W. Petty.

The President delivered an address which was very encouraging, and showed the interest he was taking in veterinary work and the advancement of the same in the State of North Carolina.

On motion, Dr. Charles H. Lockwood, B. S. Griffith, and B. E. Harper were elected members. The Secretary-Treasurer's report was read and adopted. The report on legislation showed that a hard fight had been made for our bill in the last Legislature, which only passed the Senate. The same committee was appointed to continue the work. The meeting adjourned to meet in Greensboro in December, 1897.

The second day was spent in visiting the stock- and dairy-farms and other places of interest.

J. W. PETTY,
Secretary.

